# Impact of minimally invasive mitral valve surgery on sexual dysfunction in male patients

**DOI:** 10.1186/s13019-022-01814-w

**Published:** 2022-04-14

**Authors:** Liang-liang Yan, Mi-rong Tang, Xiao-fu Dai, Liang-wan Chen, Guan-hua Fang

**Affiliations:** 1 Department of Cardiovascular Surgery, Union Hospital, Fujian Medical University, Fuzhou, China; 2 Key Laboratory of Cardio-Thoracic Surgery (Fujian Medical University), Fujian Province University, Fuzhou, China

**Keywords:** Sexual dysfunction, IIEF-5, Totally endoscopic mitral valve surgery, Quality of life

## Abstract

**Background:**

Sexual dysfunction after cardiac surgery can seriously affect patients’ quality of life, but the impact of cardiac surgery on sexual function has long been neglected. Compared with conventional cardiac surgery, minimally invasive cardiac surgery has the advantages of aesthetic appearance and no disruption of the sternal structure, which can greatly improve the patient's quality of life. However, studies focusing on the effects of minimally invasive mitral valve surgery (MIMVS) on sexual function have not been reported. The objective of this research was to investigate the effects of totally endoscopic mitral valve surgery on health-related quality of life and sexual function in male patients and to provide possible recommendations.

**Methods:**

Patients who underwent median sternotomy or totally endoscopic mitral valve surgery at our institution from January 2019 to December 2020 were selected using an electronic medical record system. Data were collected by questionnaires, including the MOS 36-item short-form health survey and the International Erectile Function Questionnaire.

**Results:**

There were 156 male patients who participated in our study. Of these, 112 patients completed all questionnaires. Forty-five patients (40.18%) developed postoperative sexual dysfunction, including 15 patients (29.41%) in the MIMVS group and 30 patients (49.18%) in the conventional MVS group, indicating that the incidence of sexual dysfunction could be reduced by MIMVS and that the MIMVS group scored better on the International Erectile Function Questionnaire (*P* < 0.05). On the evaluation of health-related quality of life, the MIMVS group scored better than the MVS group on the mental health and bodily pain subscales of the MOS 36-item short-form health survey. In addition, our study showed that postoperative sexual dysfunction was associated with physical functioning and mental health.

**Conclusions:**

In our study, totally endoscopic mitral valve surgery had less adverse effects on sexual function in male patients than conventional mitral valve surgery. In terms of health-related quality of life, totally endoscopic mitral valve surgery was superior to conventional surgery. Patients who opt for totally endoscopic mitral valve surgery may have a more satisfying and healthier sexual life than those who undergo conventional mitral valve surgery.

## Introduction

Sexual dysfunction, especially erectile dysfunction in men, is very common in patients with cardiovascular disease [1, 3]. In the past, surgeons and patients focused more on the efficacy of the procedure, and sexual dysfunction after cardiac surgery was often neglected. In recent years, as the clinical outcomes of surgery have improved, many patients have become concerned not only about the treatment itself, but also about its adverse effects on health-related quality of life (HRQoL) and even on sexual life [4–6].

It is well known that the huge trauma of median sternotomy and cardiopulmonary bypass as well as the long postoperative recovery period often cause great harm to the mental and physical health of patients, which in turn seriously affects the recovery of postoperative sexual function and even leads to postoperative sexual dysfunction [4, 5, 7, 8]. In recent years, with the development of surgical techniques, instruments and perfusion strategies, minimally invasive cardiac surgery has gradually gained widespread popularity. Minimally invasive surgery offers the advantages of less pain intensity, less trauma, smaller incisions, and faster recovery. Many literatures have shown that the clinical outcomes of minimally invasive cardiac surgery are comparable to those of conventional open surgery [9–11]. Based on the benefits of minimally invasive cardiac surgery, studies have begun to examine its impact on patients' quality of life. Previous studies have shown that HRQoL after minimally invasive cardiac surgery is better compared to conventional median sternotomy cardiac surgery [11–13].

However, studies on sexual dysfunction after minimally invasive cardiac surgery are scarce. The recovery of sexual activity is also not to be neglected as an important factor in postoperative psychosocial recovery [14, 15]. Because sexuality is also an aspect of quality of life, and a healthy and active sexual life can make an important contribution to a good quality of life [15, 16].

Although patients undergoing minimally invasive cardiac surgery can return to daily activities earlier, with less psychological impact and improved HRQoL, however, little attention has been paid to postoperative sexual function in patients undergoing minimally invasive cardiac surgery and the association between sexual function and patient HRQoL. The objective of this research was to investigate whether there are differences in sexual dysfunction and HRQoL between totally endoscopic mitral valve surgery and conventional mitral valve surgery, as well as to assess the correlation between postoperative HRQoL and sexual dysfunction.

## Materials and methods

### Patient selection

This study was designed as a single-centre, descriptive cross-sectional survey. We collected data from the electronic medical record system on male patients who underwent totally endoscopic mitral valve surgery through the establishment of cardiopulmonary bypass via the femoral artery and femoral vein or who underwent conventional MVS between January 2019 and December 2020.

Our inclusion criteria included: (1) male patients aged 18–60 years; (2) patients who were discharged from the hospital after surgery and survived for more than 6 months; (3) patients with a regular sexual partner; (4) patients who were willing to participate in this study and signed the informed consent form; and (5) patients who had no communication impairment and were able to read and understand the contents of the questionnaire.

Exclusion criteria included: (1) patients who were blind or had neurological or psychiatric disorders that prevented them from reading or writing, or had difficulty or incoordination in verbal communication; (2) patients with serious diseases of the reproductive organs or other vital organs; (3) patients with serious preoperative sexual dysfunction; and (4) patients who were on medications that severely affect sexual function.

This study protocol was approved by the institutional review board and ethics committee of Union hospital (2021KY169). All patients aged 18–60 years who underwent mitral valve surgery were eligible to participate. During this period, a total of 156 consecutive patients who underwent mitral valve surgery and met our inclusion and exclusion criteria were enrolled. Demographic characteristics included age, marital status, and smoking history. Clinical data included comorbid chronic conditions, medication history, surgical procedures, and cardiac function. We numbered the patients and then sent questionnaires to the 156 patients through telephone consultations, text messages, social networking software, or outpatient follow-up visits. Data were collected via questionnaires, and the process was completely anonymous.

### Surgical techniques

For conventional mitral valve surgery, we used the median sternotomy approach to establish cardiopulmonary bypass. The surgery was performed following a routine protocol.

Totally endoscopic cardiac surgery relies more on perfusion techniques and different anesthesia protocols. During cardiopulmonary bypass, the right lung was routinely deflated using a double-lumen endotracheal tube. External defibrillator pads and transesophageal echocardiography were also essential for cannulation of the femoral vein and artery and defibrillation.

The patient underwent a right-sided mini-thoracotomy in a supine position with the right chest elevated. The peripheral vessels were then cannulated to establish cardiopulmonary bypass. First, we made an incision in the right inguinal fold to expose the surgical area, and then we cannulated the femoral vessels under direct vision using the Seldinger-guided technique. In some cases, percutaneous puncture of the internal jugular vein may be performed if cardiopulmonary bypass flow is inadequate. Guided by transesophageal echocardiography, a single two-stage femoral vein cannula or separate superior and inferior vena cava cannula was then correctly positioned into the superior and inferior vena cava. Arterial perfusion was performed through a right femoral artery cannula. Vena cava return blood flow was usually adequate when the cannula was properly placed and vacuum-assisted venous drainage was initiated. Then, three right thoracic ports were made and soft tissue retractors were used for the protection of the incision and to prevent rib fracture during rib spreading. After that we performed aortic occlusion using a transthoracic clamp [17]. And the subsequent protocol was performed routinely [18].

Patients were given a transthoracic echocardiogram prior to discharge and were informed of outpatient follow-up visits at three months, six months, and annually postoperatively to assess physical status.

### Tools to assess sexual dysfunction

For male sexual dysfunction, we used the five-item International Index of Erectile Function Questionnaire (IIEF-5) to assess it. Erectile dysfunction is the inability of a man to achieve and maintain an adequate erection of the penis for satisfactory sexual intercourse [19]. In 1997, Rosen et al. designed the 15-item International Index of Erectile Function. It originally consisted of 15 items that measured erectile function by calculating the total score [20]. In 1998, Rosen simplified it to the IIEF-5 with five main questions to facilitate clinical application without compromising its accuracy. The IIEF-5 is highly recommended and is the most authoritative and widely used questionnaire on male sexual function worldwide. It includes five domains of sexual function: sexual desire, erectile function, intercourse satisfaction, orgasmic function, and overall satisfaction. Scores are calculated to assess erectile function [21]. The IIEF-5 is a valid and accurate screening tool for the diagnosis of erectile dysfunction and the severity of erectile dysfunction. Studies have shown that the IIEF-5 also has good validity and reliability in diagnosing erectile dysfunction in the Chinese population, with erectile function being bounded by score 21 [22]. An IIEF-5 score of > 21 is considered normal erectile function and ≤ 21 is considered erectile dysfunction.

### Tools to assess HRQoL

The MOS 36-item Short Form Health Survey (SF-36) is a widely used assessment tool for HRQoL. The SF-36 is applicable to the assessment of health in clinical populations with a variety of disorders. The questionnaire includes eight health domains: general health, mental health, role physical, physical functioning, bodily pain, vitality, role-emotional and social functioning [23, 24]. A higher score on a particular subscale indicates a higher HRQoL in that domain.

### Statistical analysis

In this study, SPSS 24.0 was used for statistical analysis, and *P* values less than 0.05 were considered statistically significant. For continuous variables with a normal distribution, values were expressed using the mean ± standard deviation and the independent samples t-test was applied for comparison, and for non-normally distributed data the Mann–Whitney U-test was used. For categorical data, the Chi-square test or Fisher's exact test was used. The Spearman's correlation test was used to assess the correlation between IIEF-5 scores and SF-36 subscale scores.

## Results

### Demographics data of the research object

The overall response rate of the study was adequate, with 124 (79.49%) participants completing the IIEF-5 and 139(89.10%) participants completing the SF-36. Of these, 112 patients (71.79%) answered all questionnaires completely and 44 patients (28.21%) did not effectively complete the questionnaire. Those who underwent totally endoscopic mitral valve surgery were included in the MIMVS group, and those who underwent conventional mitral valve surgery were included in the MVS group. There were 61 patients in the MVS group and 51 in the MIMVS group.

The age of patients who completed the questionnaire ranged from 22 to 60 years with a mean age of 45.56 ± 9.41 years, 61 patients (mean age 45.25 ± 9.05 years, 54.46%) were in the MVS group and 51 patients (mean age 45.93 ± 9.90 years, 45.54%) were in the MIMVS group. There were 87 married cases (77.68%) and 25 unmarried or divorced cases (22.32%), all of whom had regular sexual partners. There were 37 cases of smoking, 9 cases of diabetes mellitus, and 18 cases of hypertension. No difference in beta-blocker usage rate was observed between the two groups. Demographic characteristics and perioperative data are shown in Table 1.

**Table 1:** Demographic and Intra-operative data compared between two groups

Item	MVS group	MIMVS group	*P*
Sexual dysfunction	30	15	0.03
Age (years)	45.25 ± 9.05	45.93 ± 9.90	0.71
BMI (kg/m^2^)	22.53 ± 1.75	22.74 ± 1.48	0.50
Marriage	47	40	0.86
Smoke	22	15	0.46
Hypertension	8	10	0.35
Diabetes	5	4	1
β-blocker	50	39	0.47
Pre-operative NYHA (median)	II	II	
Pre-operative LVEF (%)	57.05 ± 5.86	58.30 ± 6.85	0.30
*Surgery strategy*			0.09
Mitral valve repair	35	21	
Mitral valve replacement	26	30	
Tricuspid valve plasty	20	19	0.48
Post-operative LVEF (%)	56.21 ± 5.40	55.20 ± 5.01	0.31
Post-operative NYHA (median)	I	I	
Postoperative hospital stays (days)	5.88 ± 0.94	4.87 ± 0.75	*P* < 0.001

*BMI* body mass index, *NYHA class* New York Heart Association functional classification, *LVEF* left ventricular ejection fraction

### Prevalence of sexual dysfunction

The overall sexual dysfunction prevalence rate was 40.18% (45 cases), of which the prevalence rate in the MIMVS group was 29.41% (15 cases, accounting for 33.33% of all patients with sexual dysfunction), and that in the MVS group was 49.18% (30 cases, accounting for 66.67% of the patients with sexual dysfunction). There was a significant difference in the incidence of sexual dysfunction between the two groups (*P* < 0.05) (Table 1).

### Sexual dysfunction and HRQoL, pain intensity

A follow-up questionnaire was conducted 6 months postoperatively. We used the SF-36 to assess HRQoL in both groups and the results demonstrated significant differences in the mental health and bodily pain subscales (Table 2). The mental health status was better in the MIMVS group (*P* < 0.05). Postoperative bodily pain in the MIMVS group was significantly less (*P* < 0.05). Analysis of the Spearman's correlation test showed that the IIEF-5 scores correlated with the SF-36 subscale scores. The Spearman's correlation coefficients between IIEF-5 score and the score of each SF-36 subscale were shown in Table 3. The results showed that patients with higher IIEF-5 scores also had higher scores on the SF-36 mental health and physical functioning subscales.

**Table 2:** SF-36 scores were compared between the two groups after surgery

Item	MVS group	MIMVS group	*P*
Physical functioning	78.595 ± 8.1667	77.738 ± 8.4941	0.59
Role physical	71.31 ± 15.025	72.55 ± 15.211	0.67
Bodily pain	69.84 ± 12.449	75.88 ± 16.146	0.03
General health	63.03 ± 11.339	63.82 ± 13.914	0.74
Vitality	63.03 ± 11.339	63.33 ± 12.715	0.90
Social functioning	69.34 ± 11.512	69.70 ± 12.574	0.88
Role emotional	62.52 ± 13.084	65.57 ± 16.391	0.28
Mental health	68.79 ± 17.766	75.37 ± 14.094	0.03

**Table 3:** The coefficient of rank correlation between the SF-36 scores and the IIEF-5 scores

Scale	Coefficient of rank correlation	*P* value
Physical functioning	0.869	*P* < 0.001
Role physical	0.048	0.613
Bodily pain	0.066	0.490
General health	0.020	0.832
Vitality	− 0.016	0.869
Social functioning	− 0.002	0.983
Role emotional	0.019	0.842
Mental health	0.941	*P* < 0.001

Correlation coefficients: 0–0.20 = ‘‘week’’; 0.21–0.40 = ‘‘fair’’; 0.41–0.60 = ‘‘moderate’’; 0.61–0.80 = ‘‘strong’’; 0.81–1.00 = ‘‘strongly correlation’’

## Discussion

With advances in minimally invasive surgical instruments and perfusion techniques, cardiac surgeons have begun to perform minimally invasive cardiac surgery and have devoted themselves to improving the surgical skills of minimally invasive surgery. However, surgeons and patients have long been more concerned with the clinical outcomes of minimally invasive surgery than with postoperative quality of life. In recent years, the importance of postoperative HRQoL has been gradually recognized and the impact of minimally invasive cardiac surgery on postoperative HRQoL has been studied. Studies have demonstrated that minimally invasive cardiac surgery improves postoperative HRQoL significantly compared to conventional median sternotomy approach cardiac surgery [25, 26]. But the often-overlooked fact is that sexual activity is also an important component of cardiovascular disease patients' quality of life [27]. A healthy and active sex life can make an important contribution to a good quality of life [16].

### Post-operative sexual dysfunction

Unlike the clinical outcomes of surgical treatment, resumption of sexual activity after cardiac surgery remains a neglected issue by surgeons and patients. Despite the high incidence of post-surgical sexual dysfunction, few patients choose to consult with their surgeons about when they can resume sexual activity after cardiac surgery. When patients are discharged from the hospital, cardiothoracic surgeons rarely advise patients on how to resume their sexual life after surgery. Especially in some Asian countries, such as Japan, the rate of visits for sexual dysfunction is close to zero due to the privacy of sexual life [28]. There are very few studies focusing on postoperative sexual dysfunction. There are no studies focusing on sexual dysfunction after minimally invasive cardiac surgery.

Normal sexual function is both a psychological and physiological process, and it has often been found to be related to a variety of factors, including age, gender, illness, medications, psychological state, and hormones [29]. Studies have revealed that surgery can impair sexual function in a number of different ways [30–32]. In open-heart surgery, Chen and Steinke found that concerns about possible cardiac symptoms from sexual activity, the surgical trauma, and the effects of postoperative cardiac medications can all contribute to sexual dysfunction [33, 34].

Our study showed that there was a certain incidence of sexual dysfunction after surgery (40.18%), which was 29.41% in the MIMVS group, significantly lower than in the MVS group (49.18%).

There were no significant differences in NYHA classification and LVEF values between the two groups, but the postoperative incidence of sexual dysfunction was lower in the MIMVS group than in the MVS group, which may be related to physiological and psychological factors. It may be due to the advantages of minimally invasive cardiac surgery such as: no damage to the sternum, less pain intensity, less readily visible incisions, earlier return to sexual life, and reduced incidence of sexual dysfunction.

The American Heart Association's 2012 scientific statement on sexual activity noted that minimally invasive cardiac surgery without or with partial sternotomy has the potential to allow patients to resume sexual activity sooner. Robot-assisted surgery as an iteration of minimally invasive surgery avoids sternotomy. Our totally endoscopic approach is similar to robotic surgery without disruption of the sternum, and patients who undergo this procedure may also resume sexual activity sooner than patients undergoing median sternotomy cardiac surgery [27]. However, this statement is not supported by any corresponding literature. Only one study mentioned that patients who underwent minimally invasive heart valve surgery returned to normal activity almost one month earlier than those who underwent median sternotomy cardiac surgery [12].

We believe that the reasons for the lower incidence of postoperative sexual dysfunction after MIMVS are multiple. The characteristics of rapid recovery after endoscopic surgery can promote early recovery of postoperative sexual activity and reduce the psychological impact on patients, thus reducing the incidence of post-operative sexual dysfunction. Our study showed that the incidence of postoperative sexual dysfunction can be significantly reduced in totally endoscopic MVS compared with conventional MVS based on the characteristics of minimally invasive cardiac surgery with less pain intensity, aesthetic appearance and rapid recovery.

### Sexual dysfunction and HRQoL

The American Heart Association states that sexual activity is similar to light to moderate intensity physical activity for a short period of time. The risk of adverse cardiovascular events is lower in people with a good heart function. Most patients can engage in sexual activity after thorough assessment prior to their physical condition [27].

Although physical function improved in patients after cardiac surgery (mean LVEF and cardiac function were normal in our study population). However, several studies on cardiac disease have shown that sexual dysfunction still seems to be prevalent in patients after cardiac surgery. In our study, up to 40.18% of patients were frustrated with their sexual function after surgery, and our correlation analysis also showed that patients with sexual dysfunction have a poor quality of life. The etiology of sexual dysfunction after cardiac surgery is multifactorial and includes hormonal, anatomical, physiological and psychological influences. Although death from sexual intercourse is rare in cardiac patients, a majority of patients avoid sexual activity due to fear of sudden death, anxiety, depression and decreased libido [27, 35].

According to the World Health Organization's definition of sexual health, sexual health is the integration of the physical, emotional, social, and intellectual aspects of sexual being that enriches and enhances personality, love, and communication in a positive way [36]. This definition indicates that sexual health is not just the absence of dysfunction. But we often pay attention to only limited aspects of sexual health, especially physical functioning. The recovery process after cardiac surgery requires attention not only to physical changes, but also to psychosocial adaptation [37].

The analysis of SF-36 and IIEF-5 data in this study showed that the SF-36 bodily pain and mental health subscales scores were lower in the MVS group than in the MIMVS group. In addition, we found a positive correlation between SF-36 and IIEF-5 scores, with higher scores on the physical functioning and mental health subscales being associated with higher IIEF-5 scores. This correlation suggested a significant impairment in postoperative quality of life for patients with sexual dysfunction in terms of mental health and physical functioning items, which was consistent with the change trend of quality of life after valve surgery in 2015 [8].

#### Sexual dysfunction is closely related to mental health

In terms of psychological factors, in addition to factors such as age, medications and lifestyle, research has shown that mental health is associated with sexual dysfunction. For example, a strong correlation has been found between depression and sexual dysfunction [38]. The main clinical manifestations of such patients include decreased sexual desire, erectile dysfunction and reduced sexual activity [39, 40].

Litwin et al. concluded that the mental health of patients with erectile dysfunction was significantly impaired. Specifically, they reported that low scores in mental health on the SF-36 scale were associated with more severe erectile dysfunction in male patients [41]. In addition, many studies have also shown that psychological depression is common after heart surgery [8, 42, 43].

In our study, we found that sexual dysfunction was closely related to mental health, and patients with lower IIEF-5 scores had lower mental health scores on the SF-36 scale. Our study showed that the MIMVS group had better mental health than the conventional surgery group, and the mental health score was significantly and positively correlated with the IIEF-5 score. It may be that more patients undergoing median sternotomy MVS regard sex as strenuous exercise and are concerned about its complications. For example, postoperative patients who underwent median sternotomy MVS may worry about the large unsightly scars on the chest and sexual activity affecting the healing of the sternum, and these psychological concerns can affect the sexual function of the patient, which in turn impairs mental health.

#### Sexual dysfunction is closely related to physical health

In our research, no significant differences in physical health status were observed between the two groups, indicating similar clinical outcomes for the two surgical approaches. Besides, we could also observe a correlation between sexual dysfunction and physical function subscales.

Healthy cardiac and physical function is essential for healthy sexual function [44]. Nehra indicated that both erectile dysfunction and cardiovascular disease share common risk factors, such as hypertension, diabetes, high cholesterol and smoking. Both diseases also share the same pathophysiology, mediated by endothelial dysfunction, which leads to dysfunction of the cardiovascular and reproductive organs, individually or in concert [29, 45]. In addition, a recent study identified erectile dysfunction as one of the main causes of poor adherence or cessation of cardiovascular therapy [16]. This study indicated that sexual function also affects physical health and that a healthy and active sexual life can make an important contribution to a good quality of life, thus promoting physical health.

Our study also showed that the overall pain intensity was significantly less in the MIMVS group than in the MVS group, which is coherent with previous researchs [12, 46]. Among the patients with sexual dysfunction, the pain intensity was significantly stronger than that of the patients without sexual dysfunction, and among the patients with sexual dysfunction, the pain was more intense and significantly stronger in the MVS group than in the MIMVS group. It is hypothesized that postoperative pain may be an influential factor in sexual dysfunction.

Previous studies have shown a general lack of physical activity after cardiac surgery [2, 47]. The cause of physical inactivity is unlikely to be entirely due to impaired physical functioning after cardiac surgery, since post-operative cardiac function and mean LVEF are almost normal in our study population. Physical inactivity may also be caused by psychological depression, anxiety or fear. These results of our study suggested that both physical and psychological factors have an impact on HRQoL in patients undergoing surgery, with better HRQoL in MIMVS patients than in MIMVS patients.

The literature demonstrates that minimally invasive cardiac surgery has the same surgical results as median sternotomy cardiac surgery, and that minimally invasive surgery has the advantages of faster postoperative recovery, lower pain intensity and better cosmetic appearance and without adverse effects on the patient's prognosis, thus having less impact on postoperative HRQoL compared to conventional surgery. [9–13, 25, 26].

Our study showed that minimally invasive surgery is less detrimental to post-operative sexual function and has a lower incidence of postoperative sexual dysfunction than median sternotomy MVS. Patients undergoing MIMVS may also return to normal sexual activity and improve sexual satisfaction sooner than those undergoing median sternotomy MVS due to less pain intensity and better mental health.

Our study still has some limitations. First, participants in the study were small and it was a single-centre study. Second, this study did not involve women with postoperative sexual dysfunction, and further long-term follow-up is required. Third, there was selection bias because patients were not randomly enrolled, but were surveyed through outpatient follow-up or questionnaires distributed through smartphone social software. Finally, the subjective nature of the questionnaire is also a limitation of this study.

## Conclusion

The proportion of patients presenting with sexual dysfunction after cardiac surgery was large and higher in the MVS group than in the MIMVS group. On the SF-36 subscale, totally endoscopic surgery was better than conventional mitral valve surgery in terms of mental health and bodily pain. The degree of sexual dysfunction is related with postoperative HRQoL, mainly affecting physical functioning and mental health. The degree of sexual dysfunction was associated with postoperative HRQoL, and IIEF-5 scores were positively correlated with mental and health physical subscale scores on the SF-36. In conclusion, totally endoscopic mitral valve surgery may lead to a more satisfying and healthy sex life than conventional mitral valve surgery.

## Data Availability

Data sharing was not applicable to this article, as no data sets were generated or analysed during the current study.

## Abbreviations

MIMVS: Minimally invasive mitral valve surgery
HRQoL: Health-related quality of life
IIEF-5: The International Index of Erectile Function
SF-36: The MOS 36-Item Short-Form Health Survey
BMI: Body mass index
NYHA class: New York Heart Association functional classification
LVEF: Left ventricular ejection fraction
